# De-agglomeration and homogenisation of nanoparticles in coal tar pitch-based carbon materials

**DOI:** 10.1007/s11051-016-3362-9

**Published:** 2016-02-19

**Authors:** Maciej Gubernat, Janusz Tomala, Wilhelm Frohs, Aneta Fraczek-Szczypta, Stanislaw Blazewicz

**Affiliations:** Faculty of Materials Science and Ceramics, AGH University of Science and Technology, Al. Mickiewicza 30, 30-059 Cracow, Poland; SGL Carbon Polska S.A., ul. Piastowska 29, 47-400 Raciborz, Poland; SGL CARBON GmbH, Werner-von-Siemens-Straße 18, 86405 Meitingen, Germany

**Keywords:** Carbon, Coal tar pitch, Carbonisation, Nanoparticle, Dispersants, Particle processing

## Abstract

The aim of the work was to characterise coal tar pitch (CTP) modified with selected nanoparticles as a binder precursor for the manufacture of synthetic carbon materials. Different factors influencing the preliminary preparative steps in the preparation of homogenous nanoparticle/CTP composition were studied. Graphene flakes, carbon black and nano-sized silicon carbide were used to modify CTP. Prior to introducing them into liquid CTP, nanoparticles were subjected to sonication. Various dispersants were used to prepare the suspensions, i.e. water, ethanol, dimethylformamide (DMF) and N-methylpyrrolidone (NMP).The results showed that proper dispersant selection is one of the most important factors influencing the de-agglomeration process of nanoparticles. DMF and NMP were found to be effective dispersants for the preparation of homogenous nanoparticle-containing suspensions. The presence of SiC and carbon black nanoparticles in the liquid pitch during heat treatment up to 2000 °C leads to the inhibition of crystallite growth in carbon residue.

## Introduction

Nanocomponent-containing composite materials are an area of research due to potential industrial applications. By modifying a conventional material with a nano-sized component, a new class of materials can be fabricated with enhanced physical, mechanical and chemical performance. Such materials are today replacing conventional materials in several technical and medical applications (Wang et al. 2013; Kumar et al. 2010; Maldonado-Hódar et al. 2000; Stodolak-Zych et al. 2012).The possibility of improving or modifying a desired property of a material using a nanoparticle results from its high surface energy, among other factors. A high surface energy of nanoparticles is the reason for their uncontrolled agglomeration in a liquid suspension. Such a tendency for the agglomeration of nanoparticles makes them difficult to apply, and the disintegration of agglomerates and further homogenisation are often critical steps in material processing. One of the most popular methods for the dispersal of agglomerates of nanoparticles is sonication.

Several parameters of sonication influence the disintegration of an agglomerate including sonic frequency, sonication intensity, solvent type, temperature and external pressure (Santos et al. 2009; Dreyer et al. 2010). Sonication is also used as a method for the production of graphene via sonication-assisted liquid-phase exfoliation (Ciesielski and Samori 2014).

Coal tar pitch (CTP) is one of the most economical sources (precursor) of carbon and the main binder used in the alumina and carbon/graphite industries. In the production of coke, carbon electrode material, foam and other carbon materials, such as a binder, it yields a liquid phase enabling the plasticity of a carbon mass during mixing of the components and moulding processes. For this reason, in a specified temperature range, a CTP-based binder should perform good wettability of the solid particles of filler and low viscosity. During thermal treatment, the binder is transformed into coke, creating bridges between grains and connecting them.

The direct introduction of nanoparticles to liquid CTP is associated with the possibility of the formation of agglomerates which may adversely affect the carbonisation process. The objective of the research was to determine the optimal processing parameters of the dispersion of selected nanoparticles in CTP (graphene, carbon black and nano-silicon carbide) and assess their impact on the structure of the resulting synthetic carbon materials. The influence of selected dispersants to prepare a homogeneous suspension of nanoparticles was studied. After the dispersion process, the nanoparticles were introduced into liquid CTP and the suspension was heat-treated to 2000 °C. This work investigates the conditions for obtaining a homogeneous CTP/nanoparticle suspension, before using it as a binder in the process of thermal conversion.

The presence of selected nanoparticles affects the functional properties of the carbon matrix. Graphene is growing in popularity as a nanofiller in nanocomposite technology, due to its exceptional mechanical, electrical, thermal, biological and other properties (Dreyer et al. 2010). Carbon black (CB) is a cheap carbon nanoparticle, which has the ability to enhance coking yield of carbon precursors (Zhang et al. 2012; Menendez et al. 1996). Nano-sized particles of SiC added to some types of CTP facilitate the conversion of carbon precursors into a well-ordered graphite structure (Mikociak et al. 2014). The influence of carbides as catalysts for the graphitisation process has been reported in several works (Ōya and Marsh 1982; Takenaka et al. 2008; Charon et al. 2014; Zhou et al. 2011; Kaarik et al. 2008; Ordas et al. 2014; Yi et al. 2008). The critical problem of using these nanoparticles as modifiers is their proper dispersion in CTP binders, influencing the final properties of carbon materials (Stankovich et al. 2006).

Cost effectiveness of using nanoadditives in the production of synthetic carbon and graphite materials with CTP-based binders depends on nanoparticle type and its amount. CTP is a commercially available relatively cheap raw material applied in carbon and graphite technologies. However, its thermal conversion as a binder into synthetic carbon is an energy-consuming process. Nanoparticle addition into CTP is considered, besides others, to improve energy efficiency, e.g. by a decrease of graphitisation temperature, which is one of the most energy-consuming and expensive steps in carbon technology. Therefore, by optimising processing parameters of nanoparticle-modified CTP, the amount of energy consumed in the process may be reduced. Moreover, the benefits may also result from obtaining a new and modified carbon material with better functional properties.

## Materials and methods

### Materials

Three types of nanoparticles were used to modify CTP: graphene flakes provided by Graphene Supermarket, carbon black N220 and nano-silicon carbide provided by NanoAmor. CTP and nanoparticle characteristics are shown in Tables 1 and 2, respectively. To prepare the suspensions, four types of dispersants were used: water, ethanol, dimethylformamide (DMF) and N-methylpyrrolidone (NMP).

**Table 1:** Coal tar pitch characteristics

Properties	Range	Value	Based on
Softening point—Mettler [oC]	101–105	103,3	DIN 51 920/84
Coking values—Alcan (%)	Min. 54	54,0	DIN 51 905/81
QI amount (%)	6–9	6,3	DIN 51 921/85
TI amount (%)	Min. 24	24,0	DIN 51 906/81
Ash (%)	Max. 0.5	0,130	DIN 51 922/83
Sulphur (%)	Max. 0.6	0,450	LECO

**Table 2:** Nanoparticle characteristics

Types	Graphene flakes AO3	Carbon black N220	Beta SiC
Producers	Graphene supermarket	OMSK carbon group	NanoAmor
Grain sizes	Average flake thickness: 12 nm (30–50 monolayers) Average Particle (lateral) size: ~4500 nm (1500–10000) nm.	44–500 um	45–55 nm

### Preparation of samples

#### Suspensions for sonication effectiveness tests

In order to assess the effectiveness of the de-agglomeration process of nanoparticles by sonication, the experiment was performed in two steps: the first step covered tests to determine the optimal sonication time for various dispersants and the second step analysed the influence of dispersants on disintegration.

Nanoparticle (graphene flakes, CB, SiC) suspensions were prepared using 0.1 g of nanoparticles and 50 ml of ethanol. The suspensions were sonicated for different times from 0 to 120 min, and after sonication dynamic light scattering (DLS) measurements for each suspension were made to verify particle size distribution.

Nanoparticle suspensions for verification of the impact of dispersants on the disintegration of nanoparticles were prepared in the same proportions (0.1 g of nanoparticles and 50 ml of dispersant). The influence of four types of dispersants on nanoparticle suspension’s sonication effectiveness was analysed by DLS measurements after 5 min of sonication of carbon-derived nanoparticles (graphene flakes, CB) in each type of dispersant.

#### Samples for homogenisation of nanoparticles in coal tar pitch tests

In order to characterise the homogenisation of nanoparticles in coal tar pitch matrix, the following experiments were conducted:

CTP was preliminarily ground and sieved to the fraction between 0.4 and 0.6 mm, and sonicated nanoparticles in suspension were air dried to evaporate the solvent from the suspension. Next, dry mechanical mixing of CTP powder with a nanoparticle component was applied. Both components were intensively mixed for 5 min. The mixed powders were analysed in order to assess the dispersion of the nanoparticles in CTP by scanning electron microscopy with energy-dispersive X-ray spectroscopy. This technique was used only for SiC-containing carbon samples because carbon particles (CB and graphene) in the carbon matrix are not visible.

The second method for mixing both components (CTP + SiC nanoparticles) involved the direct preparation of sonicated nanoparticles in the suspension with CTP powder. The nanoparticles were first sonicated for 5 min in suspension followed by mixing with CTP powder (fraction 0.4–0.6 mm) for 5 min. After room-temperature evaporation of dispersant, samples were analysed by SEM and  EDS.

Nanoparticle suspensions mixed with the liquid CTP were also made as the third homogenisation method. Carbon black, silicon carbide and graphene flakes in ethanol suspensions were made and sonicated for 5 min. Similar suspensions of nanoparticles with CTP powder were prepared without sonication. The prepared suspensions were directly introduced into the liquid CTP (3 % of nanoparticles) and mechanically stirred at 155 °C to evaporate ethanol. The CTP + SiC sample was analysed by SEM and EDS. Prior to the process of carbonisation, the mixed components were compression-moulded at room temperature to form pellets.

The pellets were heat-treated in a tubular furnace to 1000 °C at a rate of 1 °C/min, and then to 2000 °C at a rate of 10 °C/min under nitrogen atmosphere. As a reference sample, CTP without nanoparticles was also prepared and heat-treated under similar conditions. Samples carbonised to 2000°C were studied by Raman spectroscopy.

### Apparatus and methods

Raman spectroscopy measurements were performed using a HORIBA Labram HR spectrometer with a camera test using a laser with an excitation wavelength of 532 nm. Raman spectra analysis was performed on Fityk software version 0.9.8. PseudoVoigt function was used for the calculation of experimental spectral line shapes and the separation of complex spectral lines. D-band, G-band, D’-band and 2D-band positions and ID/IG and I2D/IG ratios were determined. Band intensities were determined from the band areas. ID/IG ratios were used for crystallite size determination according to the equation proposed by Cancado et al. (2006):

$$L_{a} = \frac{560}{{E_{L}^{4} }}\;\left( {\frac{{I_{d} }}{I_{g} }} \right)^{ - 1}$$

where EL represents the laser wave energy [eV]

Three spectra were created for each sample by taking materials from various sample sites. The characteristic values (intensities) from the Raman spectra were determined by calculating the average of the three measurements and the standard deviation.

The DLS method was carried out on a Zetasizer Nano-ZS, Malvern Instruments, connected with Zetasizer Software version 7.02. The DLS method allows for the determination of Z-average size, the values of which are strictly connected with particle size distributions (Koppel 1972; Thomas 1987; ISO 22412:2008).

Scanning electron microscopic observations were carried out on a NOVA NANO SEM 200 Microscope connected with an EDAX EDS analyser.

Sonication process of different compositions in the form of suspensions was conducted using Cole Parmer 130P with a ¼” microtip. Amplitude was established to be on the level of 80 % which corresponds to about 14 W output power. The power is dispersant type dependent and its value in various dispersants was slightly different. During sonication, the samples were cooled in a water bath.

## Results and discussion

### Effectiveness of nanoparticle de-agglomeration tests

#### Sonication time

**Fig. 1:**
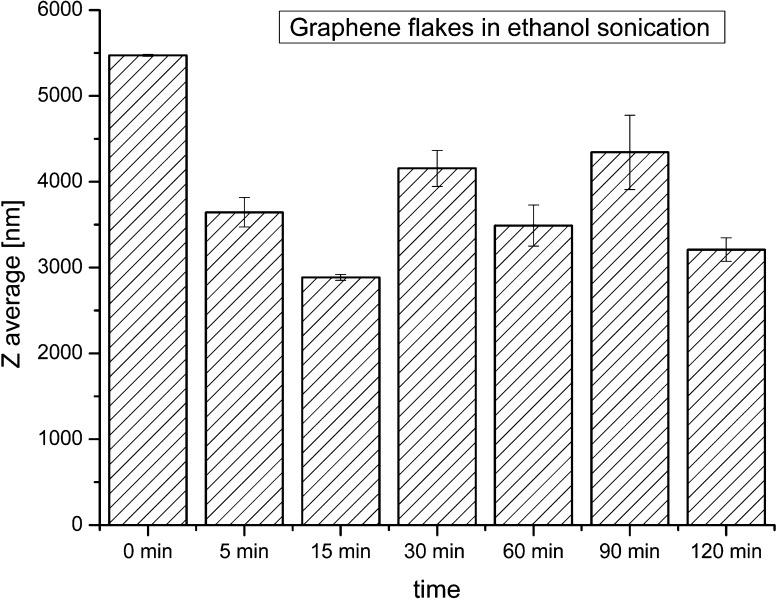
Average sizes of graphene flakes in ethanol

DLS analysis of graphene nanoflakes in ethanol (Fig. 1) showed that already after 5 min of sonication, the average zeta size decreased. Further prolongation of the process of sonication did not lead to the de-agglomeration process. Due to the irregular shapes of the graphene flakes (two-dimensional nanosheets), various large particles (average zeta sizes) were found after longer sonication time. The results indicate that the flakes are stacked to each other, and the prolongation of sonication in ethanol is not effective to receive better dispersion.

**Fig. 2:**
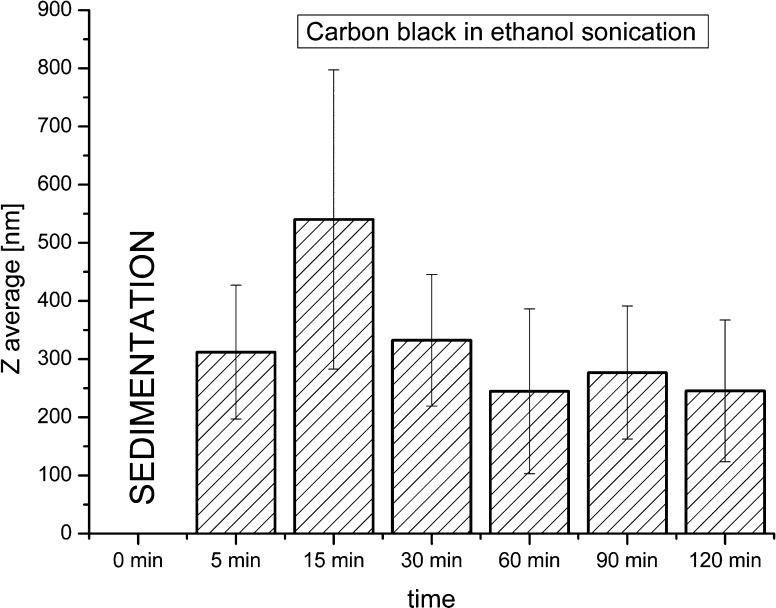
Average sizes of carbon black in ethanol

Carbon black absorbs moisture from the air and creates agglomerates that are round in shape. Due to the sedimentation process of CB aggregates, it was not possible to measure their size before sonication. Already after 5 min of sonication, nanoparticles with an average size of 300 nm were found, without further changes as sonication was prolonged. The diagram of average nanoparticle sizes after sonication for different times is shown in Fig. 2.

**Fig. 3:**
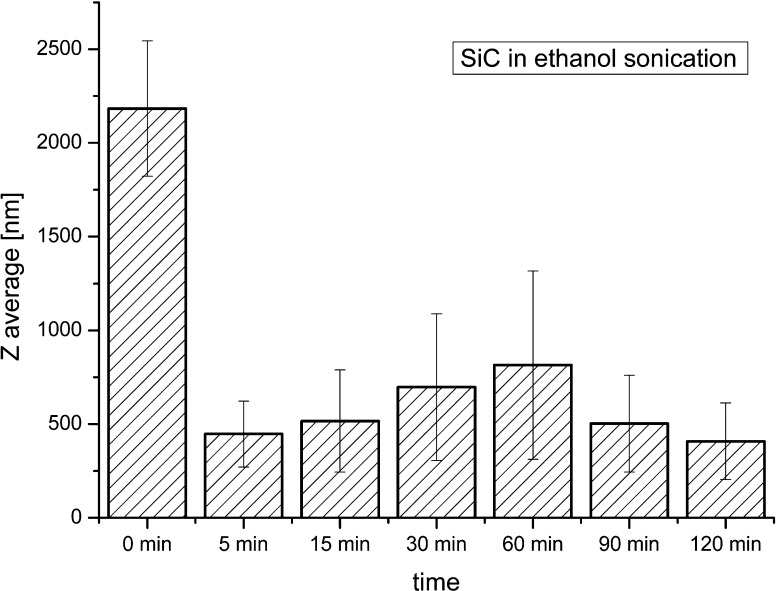
Average sizes of silicon carbide in ethanol solution

As shown in Fig. 3, sonication for 5 min is sufficient to obtain the effective disintegration of SiC agglomerates in ethanol suspension. However, according to the datasheet given by the producer, SiC nanoparticles should have an average zeta size of 45–55 nm (TEM observations),whereas the average values obtained in this work are about 400 nm, i.e. about ten times higher.

Prolongation of sonication of all nanoparticles studied in ethanol showed that this processing factor is not critical for de-agglomeration. To avoid the presence of agglomerates in the CTP matrix, the de-agglomeration by sonication is necessary.

#### Dispersants

**Fig. 4:**
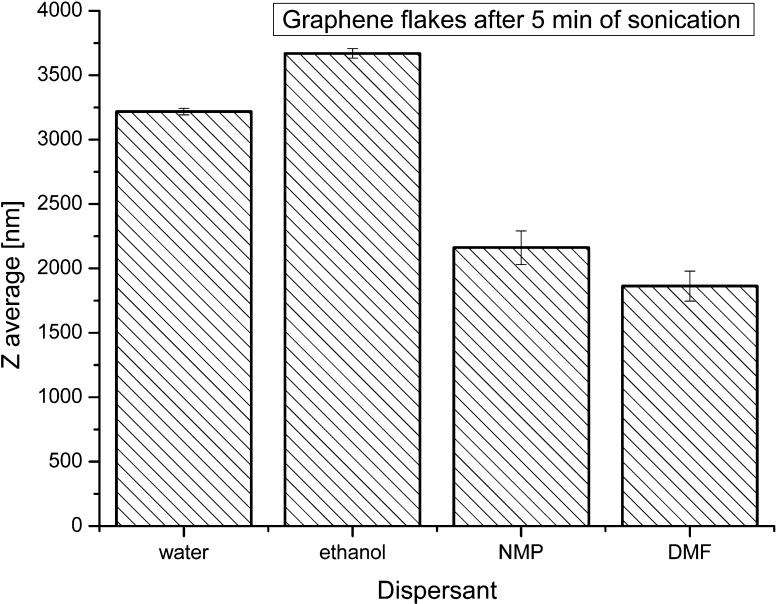
Average sizes of graphene flakes in various dispersants

Figure 4 compares the Z-average size of graphene flakes obtained in various dispersants. Graphene flakes, after 5 min of sonication in water and in ethanol, created nanoparticle sizes ranging from 3250 to 3750 nm. These values are significantly higher compared to the graphene sizes obtained with inorganic solvents. The best results were received in DMF dispersant in which the Z-average size of nanoparticles after 5 min of sonication was about 1750 nm (Fig. 4).

**Fig. 5:**
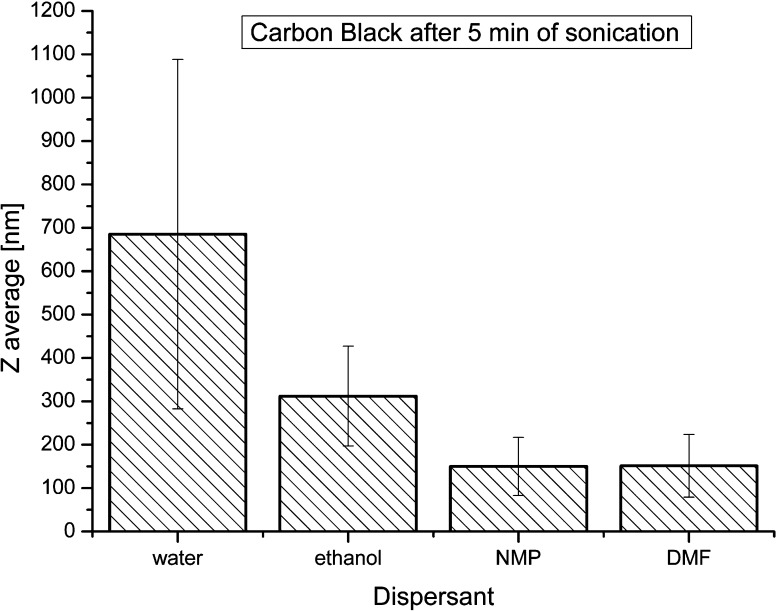
Average sizes of carbon black in various dispersants

DLS diagram of carbon black suspensions is shown in Fig. 5. These results indicate that NMP and DMF dispersants are more effective than water or ethanol. After 5-min sonication in NMP and DMF, the Z-average size of carbon black particles was about 150 nm, where, after the same time of sonication in water, the Z-average size was 700 nm (at ±400 nm SD) and in ethanol 300 nm(at ±100 nm SD) (Fig. 5).

The differences in disintegration process can be explained by differences in dispersant surface tensions. According to the literature, suitable dispersants for carbon nanoparticle sonication should have a surface tension of about 40 mJ m^−2^ (Ciesielski and Samori 2014). The surface tensions of dispersants used in this study, i.e. water at 20 °C, ethanol (CAS Ref. No.64-17-5), NMP (CAS Ref. No.872-50-4) and DMF (CAS Ref. No. 68-12-2), are as 72,80 mJ m^−2^, 22,10 mJ m^−2^, 40,79 mJ m^−2^ and 37,10 mJ m^−2^, respectively. Thus, NMP and DMF dispersants display the optimal surface tensions. However, both dispersants are known to be toxic (Ciesielski and Samori 2014). Thus, further investigation should concentrate on the chemical modification of the non-toxic dispersants including water to adjust their surface tension level during the preparation of carbon-derived nanoparticle suspensions followed by sonication processes.

### Homogenisation of nanoparticles in coal tar pitch methods

#### Mechanical dry mixing with CTP powder

**Fig. 6:**
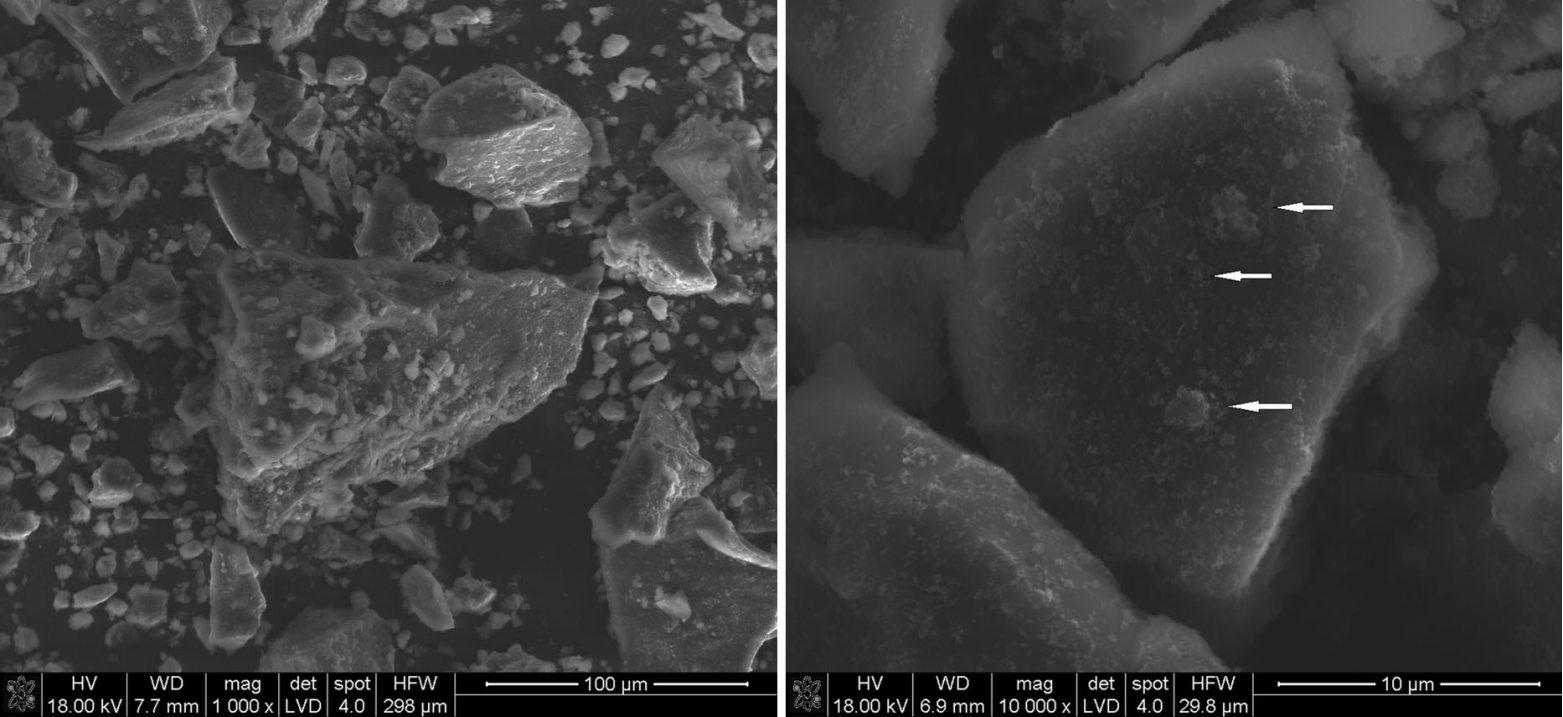
SEM of CTP + SiC nanoparticles after mechanical dry mixing; the *arrows* show aggregates formed due to secondary agglomeration

**Fig. 7:**
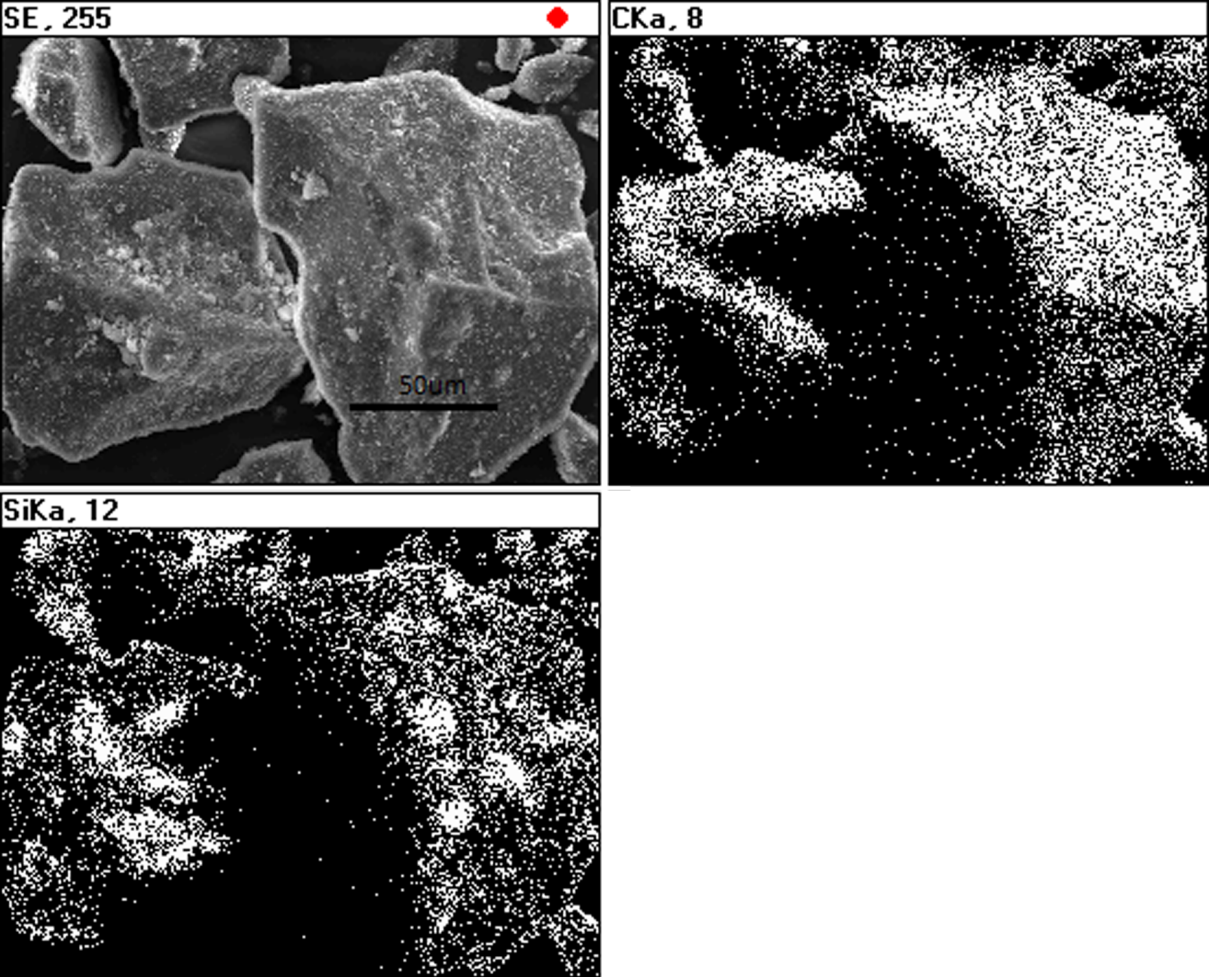
EDS mapping of CTP + SiC nanoparticles after mechanical dry mixing

Figure 6 illustrates CTP powder-containing SiC nanoparticles after mechanical dry mixing. EDS map analysis indicates that CTP grains are well covered with SiC nanoparticles (Fig. 7). However, the mixed powder composition also contains agglomerates. On the surfaces of CTP grains, SiC nanoparticles smaller than 1 µm can be found, between which bigger agglomerates are also visible (Fig. 6—shown by arrows). It is likely that such agglomerates were formed during solvent evaporation. Their sizes were around a few micrometres. After sonication, an average grain size of about 400 nm was detected (Fig. 3).

#### Mixing sonicated nanoparticle/ethanol suspension with CTP powder

**Fig. 8:**
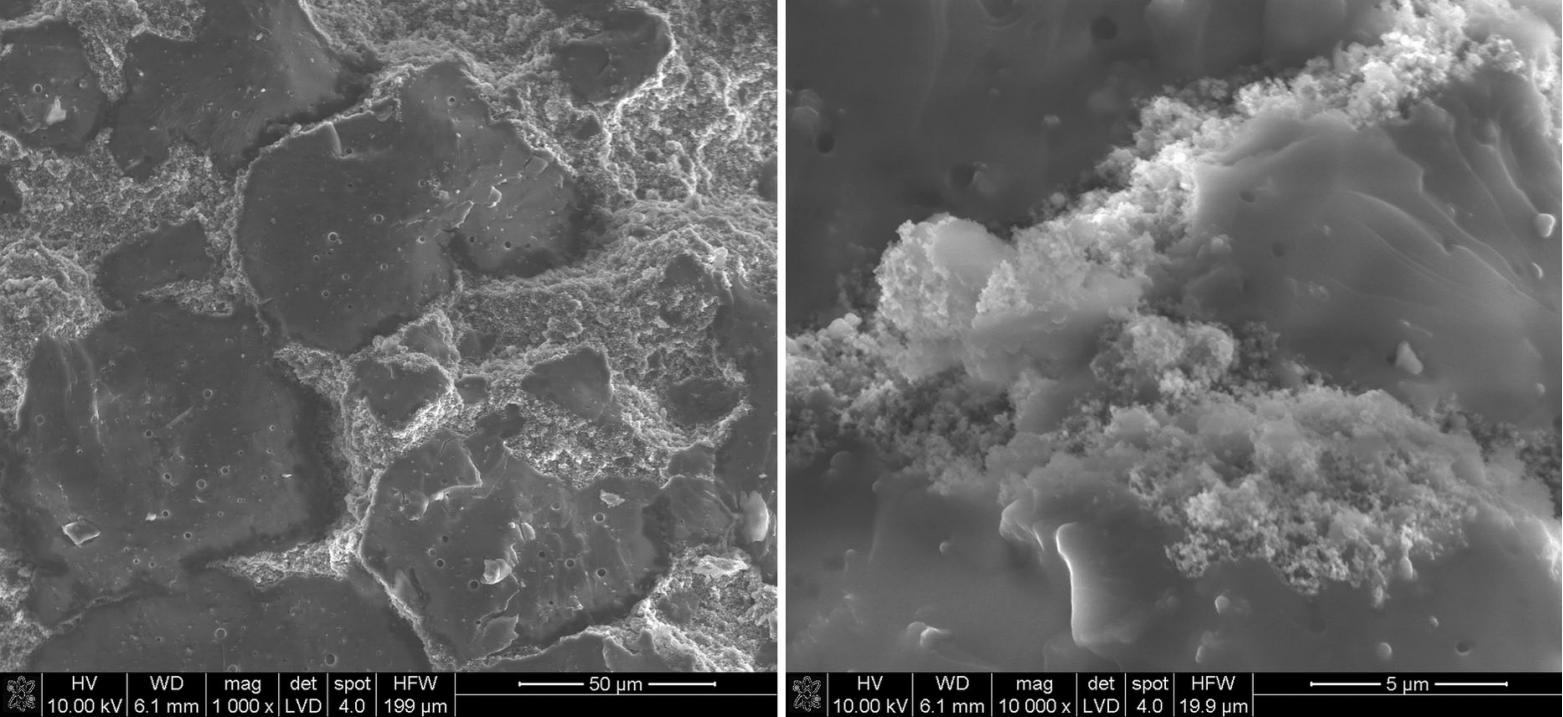
SEM of CTP powder + SiC nanoparticle suspensions after solvent evaporation; *arrows* show the CTP grain surface covered with SiC nanoparticles

**Fig. 9:**
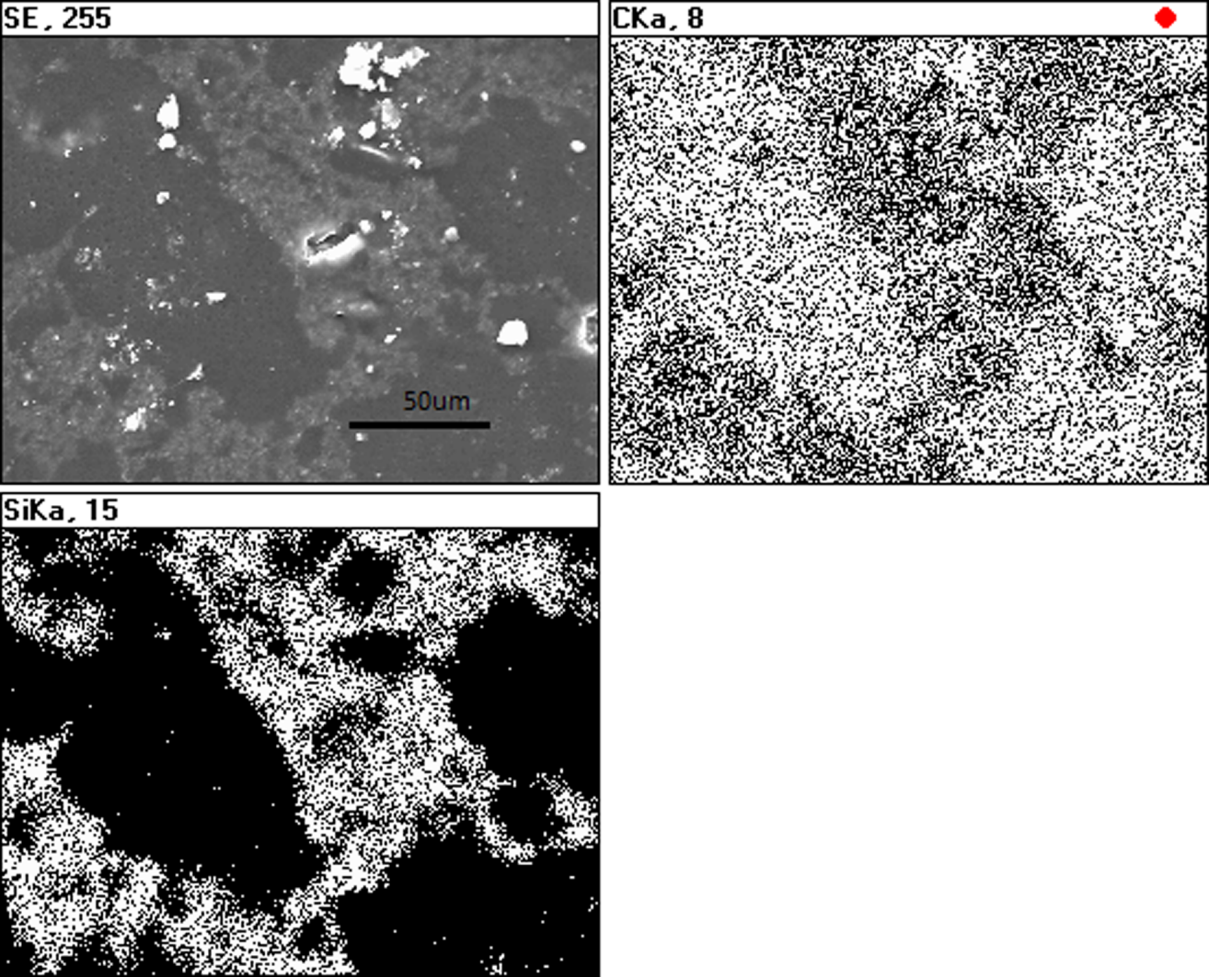
EDS mapping of CTP powder + SiC nanoparticle suspensions after solvent evaporation

The effect of CTP powder mixed with ethanol nanoparticle suspensions is somewhat different compared to direct mixing of CTP powders with nanoparticles. The presence of ethanol causes a partial dissolution of CTP grain surface; after its evaporation, nanoparticles are irregularly distributed on CTP grain surfaces (Fig. 8). It is also proved by EDS map analysis (Fig. 9).

Both methods, i.e. direct dry mixing of nanoparticles and mixing of nanoparticles suspended in ethanol with CTP powder, do not effectively disperse nanoparticles within the CTP grains.

#### Mixing ethanol suspension of sonicated nanoparticles with liquid CTP

**Fig. 10:**
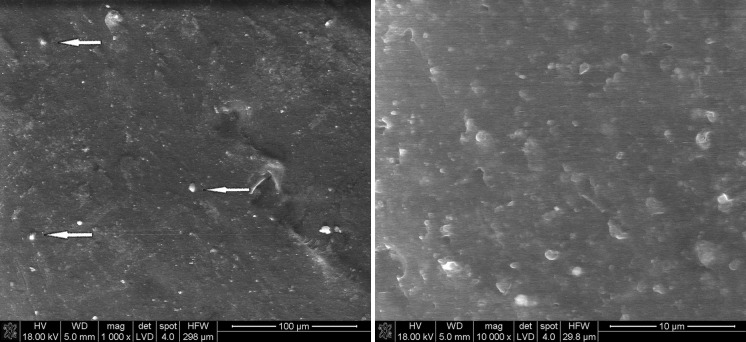
SEM of CTP + SiC nanoparticle suspension after mixing at 155 °C. *Arrows* indicate sites of SiC nanoparticles in the CTP sample

Mixed suspensions of sonicated nanoparticles with liquid CTP enable the introduction of nanoparticles in the whole volume of CTP matrix. Results (Fig. 10) indicate that SiC nanoparticles were present not only in the surface sample but also in the whole volume of CTP.

**Fig. 11:**
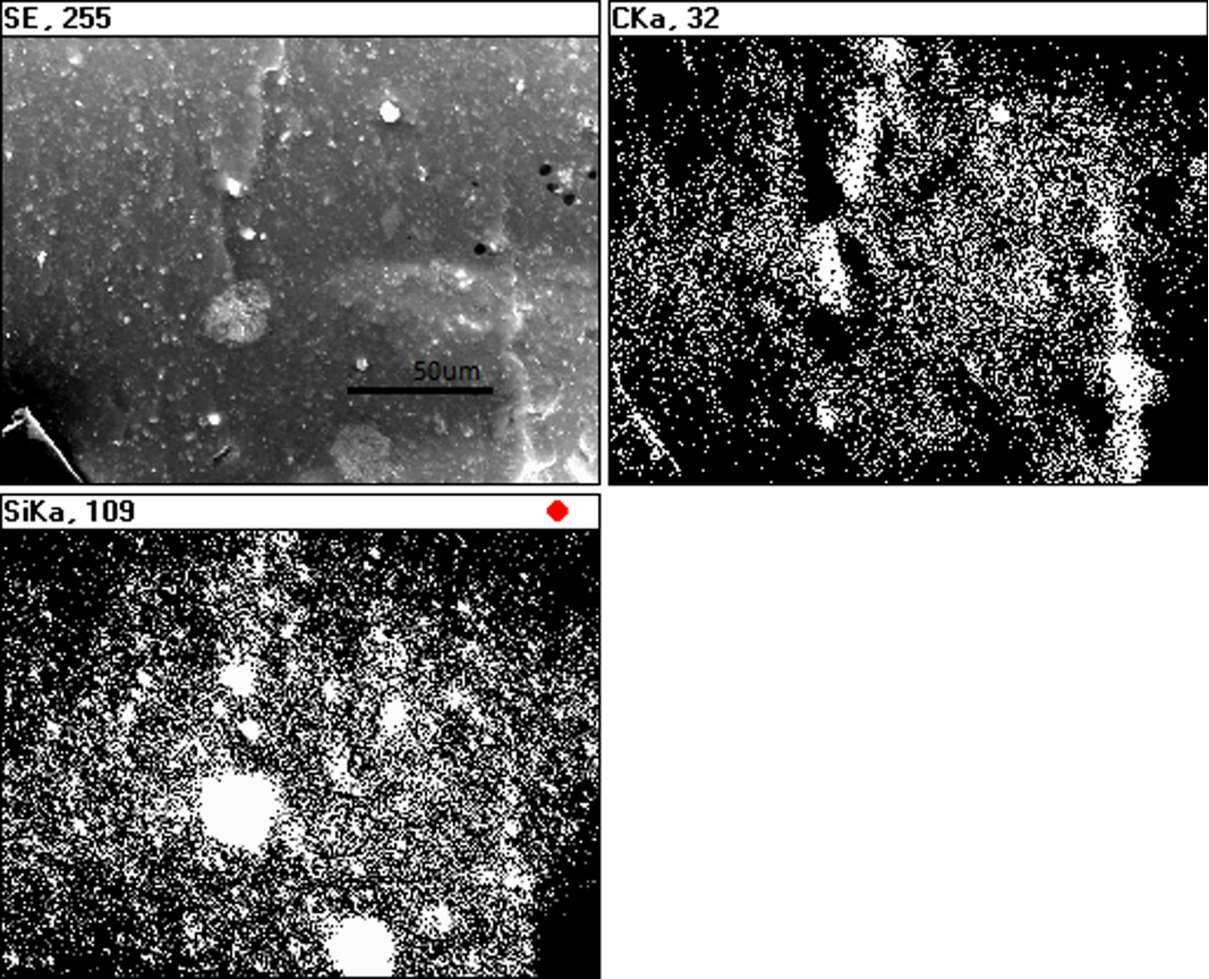
EDS mapping of CTP + SiC nanoparticle suspension after mixing at 155 °C

**Fig. 12:**
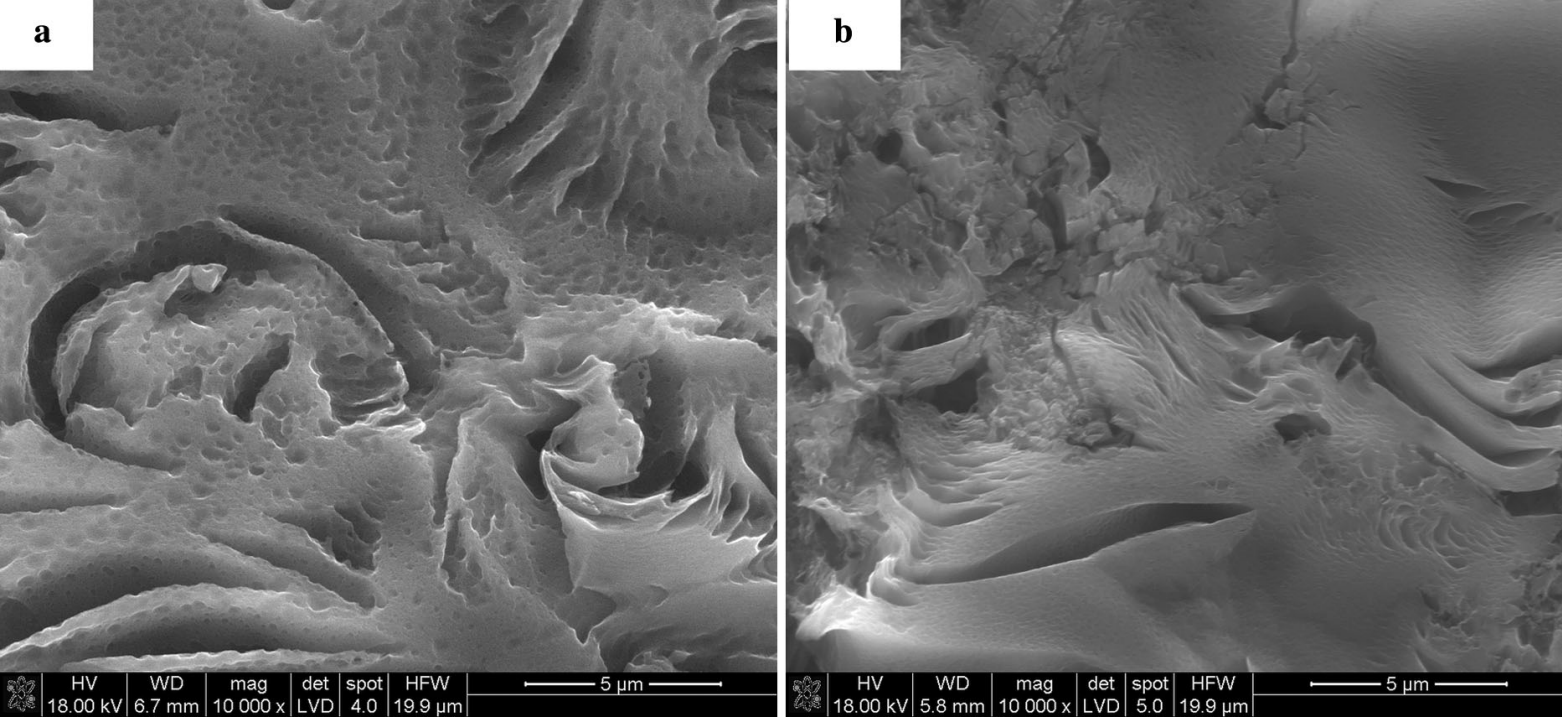
**a** SEM of CTP-based carbon residue and **b** CTP carbon residues containing graphene flakes

#### Characterisation of samples heat-treated to 2000 °C by Raman spectroscopy

**Fig. 13:**
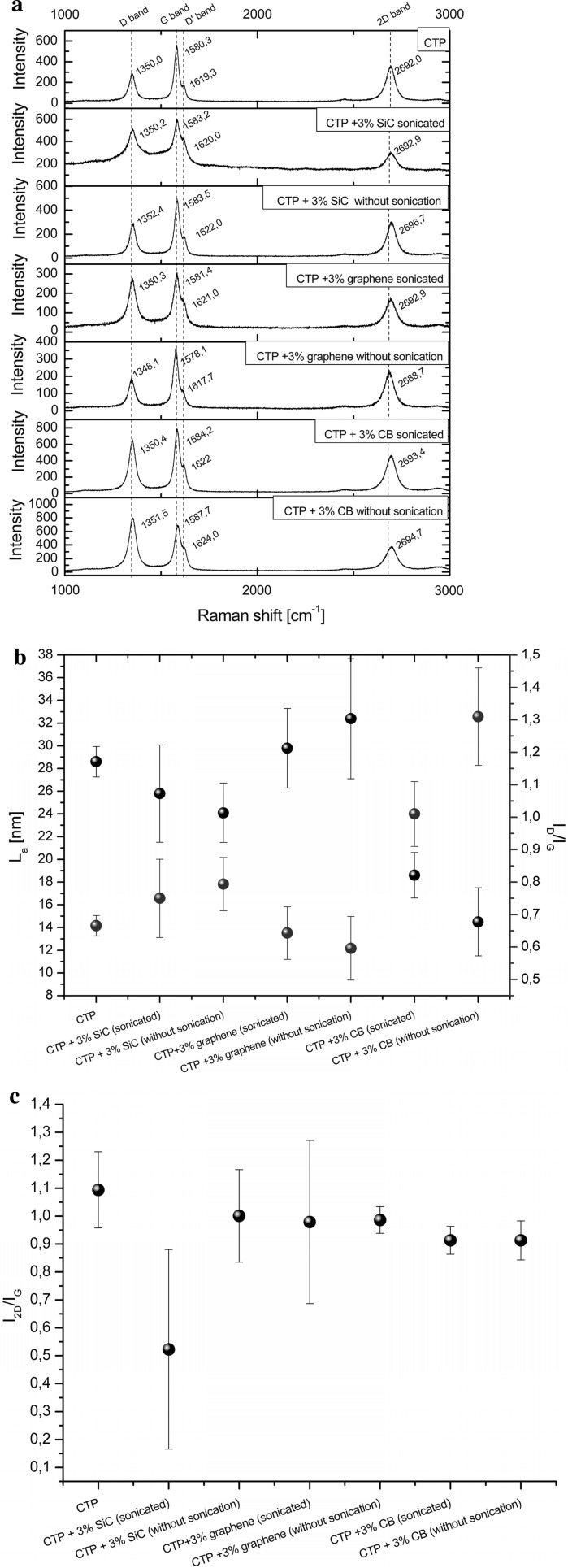
**a** Raman spectra of carbon samples obtained at 2000 °C. **b** Crystallite sizes (La) and I_D_/I_G_ ratios for CTP and CTP modified with nanoparticles after heating to 2000 °C and **c** I_2D_/I_G_ ratio for CTP and CTP modified with nanoparticles after heating to 2000 °C

It is known from the literature that there are several Raman bands found in carbonaceous substances depending on the level of carbonisation and graphitisation. The Raman spectra of pure CTP-based carbon residue and carbon compositions containing various nano-sized species are shown in Fig. 13a. SEM microstructure of CTP-based carbon residue is shown in Fig. 12a. The microstructure images of carbon residues containing nanoadditives (Fig. 12b.) are similar for all examined samples. On the contrary, differences can be observed in their structural parameters (Fig. 13). All samples containing nanoparticles were prepared by the third procedure, i.e. mixing ethanol suspension of sonicated nanoparticles with liquid CTP. The samples in the form of pellets were then heated to 2000 °C. The figure also compares the spectra of carbon compositions prepared in different ways, i.e. nanoparticles with and without sonication in liquid CTP. The spectra exhibit the distinct bands at around 1350 cm^−1^, 1580 cm^−1^, 1620 cm^−1^ and 2690 cm^−1^. Raman intensity for D-band (1350 cm^−1^), D’-band (1620 cm^−1^) and 2D-band (2690 cm^−1^) increases when the amount of disordered carbon in the material is higher. The bands are also broader, with higher FWHM values. The band at 1350 cm^−1^, known as the D-band (the A1 g mode), corresponds to imperfections or loss of hexagonal symmetry in the carbon structure. This band is also associated with the vibration of carbon atoms with dangling bonds in plane terminations of disordered carbon structures. For not well-graphitised carbon structures, the band at 1620 cm^−1^ is present; this is known as the D’-line and is a shoulder of the G-line. It can be attributed to the carbon phase, representing a less-ordered carbon structure when material is composed of defected nanocrystalline graphite. For a highly disordered structure, with broadening of the G and D’ bands, it is convenient to consider a single G-line. The average G-band positions then move from ~1580 to ~1600 cm^−1^. Raman scattering from a well-crystallised graphite structure is limited to a single peak centred at about 1580 cm^−1^, the so-called E2 g or G-band (Marella and Tomaselli 2006; Ferrari 2007).

The band at 2690 cm^−1^, known as the 2D-band, corresponds to a second-order D-band. In the case of the turbostratic carbon structure, i.e. with disturbed AB planar stacking, the Raman spectrum exhibits a single 2D-band (Ferrari 2007). It is known that a symmetric single peak at 2690 cm^−1^ refers to few graphene layers. Band intensity values as ID/IG and I2D/IG ratios and an La parameter determined from Cancado formulae were used for a more detailed comparison of carbon structure and for analysing samples and differences between them. The intensity ratio of D- and G-bands, ID/IG, is a measure of disorder degree in the carbon structure. Moreover, I2D/IG ratio allows the number of stacked graphene layers, also known as “a crystallite height”, to be roughly established, whereas La denotes the crystallite thickness in the carbon structure.

The comparison of band intensities related to ordered and disordered carbon components in the samples after heat-treated to 2000 °C indicates that the kind of nanoparticles and their dispersion influence CTP matrix conversion into the carbon phase.

**Fig. 14:**
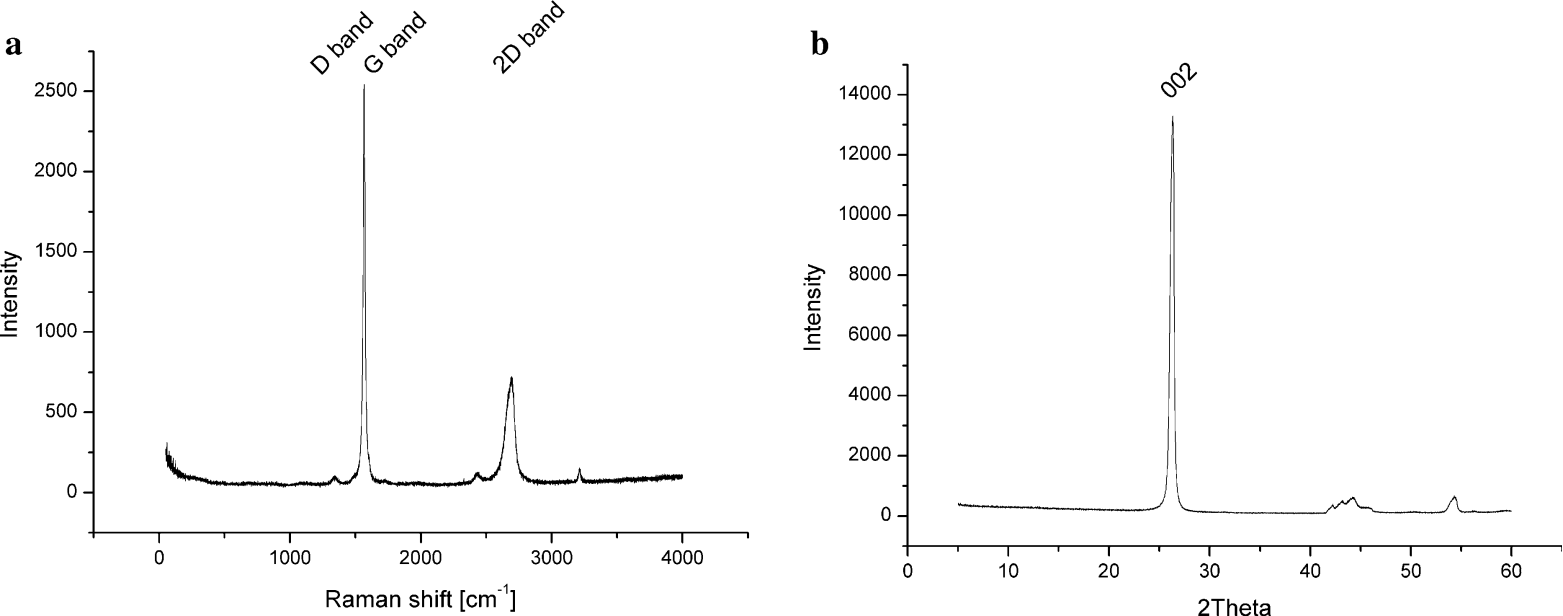
**a** Raman spectra of graphene flakes and **b** X-ray diffraction pattern of graphene flakes

The ID/IG ratio for CTP-derived carbon residue and graphene-containing CTP heated to 2000 °C is distinctly lower than that for carbon residue obtained from SiC-modified CTP and CB-modified CTP (Fig. 13b). It indicates that both nanoparticles (SiC and CB) present in CTP inhibit, to a certain extent, structural ordering of the carbon phase during carbonisation. It also cannot be excluded that small particles in the liquid pitch can act as nucleation sites during the process of pyrolysis and carbonisation resulting in the formation of a finer crystallite microstructure of the carbon phase. Graphene flakes represent a well-ordered graphitic structure with ID/IG = 0,092, La = 210 nm (Fig. 14a) and d002 = 0338 nm (Fig. 14b). Their presence in CTP during heat treatment causes structural changes of carbonised composite matrix. CTP modified with graphene and carbon residue shows a better order of the structure compared to other samples. The ID/IG ratio for this sample obtained without sonication amounts to 0.6, which is much lower in comparison to CTP-derived carbon residues modified with CB without sonication (ID/IG = 1.32).The high value of ID/IG ratio in CB-containing carbon residue indicates that disordered carbon component dominates. In the Raman spectrum of this sample, a small shoulder of the G-line and an overtone of the D-line at 2688.7 cm^−1^ can be detected. The highest crystallites were found for CTP + 3 % graphene-derived carbon samples (La about 32 nm), among all investigated samples, whereas the lowest ones were in samples containing CB nanoparticles without preliminary sonication (La = 15 nm). The influence of sonication on the nanoparticles’ ability to enhance the conversion of CTP into the graphitic structure was also confirmed in other samples; namely, for CTP samples with SiC and CB the average size of crystallites was larger after the sonication of nanoparticles compared to samples that were not sonicated. However, such dependence was not observed in the case of CTP-derived carbons modified by graphene. Due to the specific shape of graphene flakes, their dispersion within the CTP matrix is more complex than the dispersion of SiC and CB. These nanoparticles have a tendency to overlap and cerate parallel fakes, which makes their homogenisation in the liquid phase difficult. Homogenisation of round SiC and CB nanoparticles is easier. Despite lower homogenisation of graphene flakes, these particles were found to have a strong influence on the process of structural ordering of the carbon phase during thermal conversion of coal tar pitch.

As can be seen from Fig. 13b, by comparing the standard deviations of modified and unmodified samples, the crystallite size (La) distributions were much higher for the CTP samples with nanoparticles. Raman spectra made from different sites of the sample revealed the differences in crystallite sizes of the CTP matrix; bigger carbon crystallites were found in the presence of graphene as compared to the sites without graphene. Such an effect was not observed in CTP-derived carbon which was homogenous, and the distribution of crystallite sizes in the matrix was smaller.

Figure 13c shows I2D/IG ratio for all samples. The values are in the range from 0.55 to 1.12. This parameter allows the approximate number of stacking layers in crystallite to be specified. As is apparent, all samples consist of a few stacking layers (three and more), which indicates a nanocrystalline structure of ordered carbon residues obtained at 2000 °C (Si et al. 2012). A significant distribution of SD values for carbon samples obtained from Si-contained CTP indicates a non-homogenous microstructure.

## Conclusions

Prolongation of the sonication process did not alter the nanoparticle agglomerate average size. Within a few minutes, sonication gave a similar de-agglomeration effect after 120 min of sonication.

This study showed that the selection of an optimal solvent during sonication stage is more effective in the dispersion process of agglomerated nanoparticles than the sonication process. The surface tension of the optimal solvents for carbon nanoparticles (graphene, CB) amounts to about 40 mJ/m2. Dimethylformamide (DMF) and N-methylpyrrolidone (NMP) are known to be toxic substances; for that reason, further study on the selection of other additives allowing for the modification of water surface tension to an appropriate value is needed.

Prior to homogenisation, nanoparticles should be sonicated. The direct addition of nanoparticles in the form of suspension to the CTP powder causes the secondary agglomeration of nanoparticles resulting in the lowering of homogeneity of compositions. Sonicated nanoparticles should be mixed with CTP in its liquid state which enables the best homogeneity to be achieved.

Raman spectroscopy revealed that samples with carbon black and silicon carbide nanoparticles after sonication had a more ordered microstructure than the samples with carbon black without sonication. The greatest impact on the microstructure of CTP-derived carbon phase was due to the addition of graphene flakes to the CTP. This nanocomponent, due to its specific flat shape, is difficult to disperse well in liquid CTP.
